# Suppression of CYP2C9 by MicroRNA hsa-miR-128-3p in Human Liver Cells and Association with Hepatocellular Carcinoma

**DOI:** 10.1038/srep08534

**Published:** 2015-02-23

**Authors:** Dianke Yu, Bridgett Green, April Marrone, Yongli Guo, Susan Kadlubar, Dongxin Lin, James Fuscoe, Igor Pogribny, Baitang Ning

**Affiliations:** 1 National Center for Toxicological Research, US Food and Drug Administration, Jefferson, AR 72079, USA; 2 State Key Laboratory of Molecular Oncology and Department of Etiology & Carcinogenesis, Cancer Institute and Hospital, Chinese Academy of Medical Sciences and Peking Union Medical College, Beijing, China 100021; 3 Beijing Children's Hospital, Capital Medical University, Beijing, China 100045; 4 University of Arkansas for Medical Sciences, AR 72205, USA

## Abstract

Published studies have identified genetic variants, somatic mutations, and changes in gene expression profiles that are associated with hepatocellular carcinoma (HCC), particularly involving genes that encode drug metabolizing enzymes (DMEs). CYP2C9, one of the most abundant and important DMEs, is involved in the metabolism of many carcinogens and drugs and is down-regulated in HCC. To investigate the molecular mechanisms that control CYP2C9 expression, we applied integrative approaches including *in silico*, *in vitro*, and *in vivo* analyses to elucidate the role of microRNA hsa-miR-128-3p in the regulation of CYP2C9 expression and translation. RNA electrophoresis mobility shift assays demonstrated a direct interaction between hsa-miR-128-3p and its cognate target, the *CYP2C9* transcript. Furthermore, the expression of a luciferase reporter gene containing the 3′-UTR of *CYP2C9* and the endogenous expression of *CYP2C9* were suppressed by transfection of hsa-miR-128-3p. Importantly, chemically-induced up- or down-regulation of hsa-miR-128-3p correlated inversely with the expression of *CYP2C9*. Finally, an association analysis revealed that the expression of hsa-miR-128-3p is inversely correlated with the expression of *CYP2C9* in HCC tumor tissues. Altogether, the study helped to elucidate the mechanism of *CYP2C9* regulation by hsa-miR-128-3p, and the inverse association in HCC.

Primary liver cancer is the fifth most common cancer and third leading cause of cancer-related death worldwide; hepatocellular carcinoma (HCC) is the predominant subtype, accounting for approximately 85%–90% of total primary liver cancer cases^1^. Epidemiological studies have revealed several risk factors associated with the etiology of HCC, including geographic region, socio-economic status, gender, ethnicity and environmental exposures^1^. It is well documented that hepatitis B virus (HBV) or hepatitis C virus (HCV) infection, dietary aflatoxin B_1_ contamination, chronic alcohol abuse and tobacco consumption, lack of dietary antioxidants, environmental arsenic exposure, obesity, and non-alcoholic fatty liver disease are major risk factors for HCC^2^. These findings indicate that chronic xenobiotic stress plays a pivotal role in the development of HCC, although the precise molecular mechanisms are poorly understood.

During the past decades, advancing technologies, such as high-throughput microarray and next-generation sequencing, have led to the exploration of the relationship between environmental stress and cancer susceptibility by identifying genetic variants, somatic mutations, gene expression profiles and specific xenobiotic exposures associated with cancer etiologies^3^. For example, it is now thought that the major function of drug metabolizing enzymes (DMEs) is the regulation of cell growth, apoptosis, differentiation and homeostasis, while approximately 1% of their function is devoted to the metabolism of xenobiotics^4^. Genetic polymorphisms in DME genes are convincingly found to be associated with risk of toxicity and/or cancer^5^,^6^, including the association with the risk of HCC^7^. Studies also have shown that dysregulation of DMEs, including several cytochrome P450 (CYP) family genes such as *CYP2A6, CYP2C9, CYP2E1, CYP3A5*^8^,^9^,^10^, might play important roles in the development of HCC.

The human CYP enzyme superfamily is comprised of at least 57 distinct genes in 18 families that are classified as drug/xenobiotic metabolizing enzymes due to their pivotal role in protecting the organism from xenobiotics and environmental toxins by metabolizing them into inactive compounds. However, CYP450 enzyme activity also catalyzes the formation of reactive metabolites that cause DNA, RNA or protein damage^11^. Alteration in the expression *CYP* genes can affect the efficiency of xenobiotic detoxification and also the production of messenger molecules that regulate downstream signal-transduction pathways, thus having a paradoxical impact in carcinogenesis^11^. Therefore, it is reasonable to speculate that the dysregulation of *CYP* family genes might be involved in hepatocarcinogenesis. CYP2C9, one of the most abundant CYP2C proteins, accounts for ~20% of hepatic CYP content and contributes to the metabolism of many carcinogens and drugs^12^,^13^. Suppression of *CYP2C9* expression has been reported as a biomarker of HCC^14^,^15^,^16^,^17^; however, the mechanism of *CYP2C9* dysregulation through microRNA (miRNAs) modulation has not been investigated in HCC.

The miRNAs are small RNA molecules that modulate gene expression through translational repression of cognate mRNA targets and thus are important mediators in gene regulatory networks. Consequently, miRNA-dependent modulation of the expression of DMEs among individuals could lead to substantial changes in phenotypes. Such changes may have a significant influence on quantitative traits, including the development of cancer and other diseases^18^. In the last decade, many human DMEs and nuclear receptors have been reported to be regulated by miRNAs, such as CYP1B1, CYP2E1, CYP3A4, CYP 24A1, SUL1A1, PXR and VDR^19^. The miRNA-dependent regulation of DMEs and transporters should have potential effects in modulation of carcinogen metallization-either activation or detoxification, thus may be associated with cancer development. For example, evidences demonstrated that CYP1B1 is regulated by miR-27b, and a decreased miR-27b expression is inversely associated with an increased protein level of CYP1B1 in breast cancer patients^20^. Pathogenically, although the high level of CYP1B1 may decrease the estrogen activity by CYP1B1-mediated 4-hydroxylation, the increased metabolite 4-hydroxyestadiol level may contribute more significantly to breast carcinogenesis^19^.

There are several hundred miRNAs expressed in human tissues, and characterization of miRNA expression patterns in human tumor tissues reveal miRNA signatures that are associated with tumor initiation, progression metastasis, diagnosis, prognosis, response to treatment and survival^21^; however, there is a lack of conclusive information on the role for miRNA modulation of drug metabolizing genes in the pathogenesis of HCC.

In the current study, we hypothesized that hsa-miR-128-3p^22^,^23^,^24^ and hsa-miR-143-3p^25^,^26^, which play important roles in cancer, could influence *CYP2C9* expression by targeting its mRNA transcripts based on *in silico* analyses of putative mRNA/miRNA complexes. To test this hypothesis, we employed a series of biochemical assays to investigate the interaction of these two miRNAs with the *CYP2C9* transcript and identified hsa-miR-128-3p as an efficient suppressor of *CYP2C9* expression.

## Results

### Bioinformatic identification of candidate miRNAs targeting *CYP2C9*

**Figure 1:**
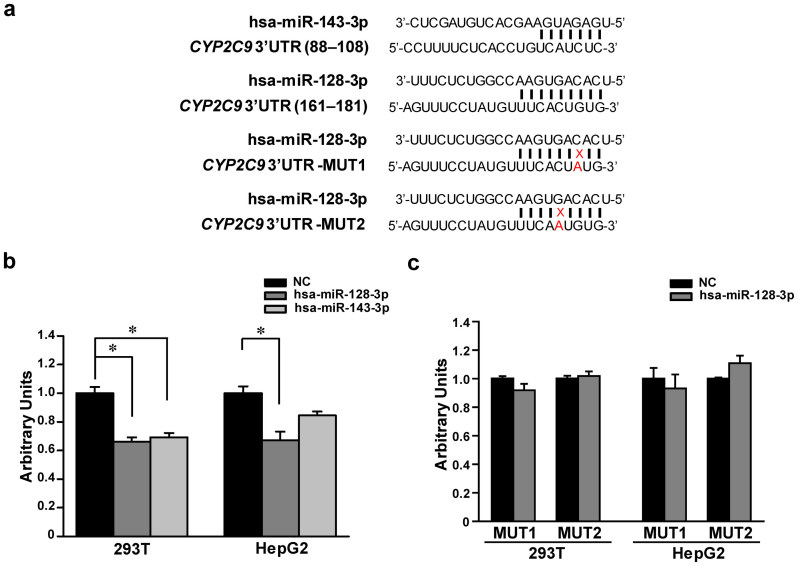
The hsa-miR-128-3p and hsa-miR-143-3p suppressed reporter gene expression. (a) Prediction of hsa-miR-143-3p targeting to the 3′-UTR of *CYP2C9*, and hsa-miR-128-3p targeting to the 3′-UTR of *CYP2C9*, as well as the “targeting” of hsa-miR128-3p to two mutated 3′UTR sequences of the *CYP2C9* gene. The solid vertical line indicates base pairing and the numbering used for the hsa-miR-128-3p and hsa-miR-143-3p target sequences (88–108 or 161–181, respectively) is consistent with that given for the *CYP2C9* 3′-UTR in NM_000771. (b) Luciferase reporter assays to investigate the effects of the hsa-miR-128-3p and hsa-miR-143-3p on *CYP2C9* 3′-UTR. (c) Luciferase reporter assays to investigate the effect of hsa-miR-128-3p on mutated CYP2C9 3′-UTRs. 293T and HepG2 cells were transiently transfected with the CYP2C9- CU, CYP2C9-MUT1-CU (containing a mutated sequence in the 3′UTR of *CYP2C9*), or CYP2C9-MUT2-CU (containing another mutated sequence in the 3′-UTR of *CYP2C9*) plasmid, together with 50 nmol/L hsa-miR-128-3p mimic, hsa-miR-143-3p mimic, or miRNA negative control, respectively, and harvested 48 hours after transfection. *Renilla* luciferase activity was measured, and then normalized to firefly luciferase. Three independent transfection experiments were carried out, and each was performed in triplicate. Data are shown as relative activity to luciferase activity expressed by the CYP2C9-CU plasmid transfection together with miRNA negative control. * *P* < 0.05; NC, miRNA negative control.

Three public databases, microRNA.org, PITA and TargetScan, applying different evaluation criteria, were used to screen for miRNAs with the potential to bind the 3′-UTR of *CYP2C9*. Several miRNAs, including hsa-miR-128-3p and hsa-miR-143-3p, were identified in all three databases. Due to the reported roles of hsa-miR-128-3p^22^,^23^,^24^ and hsa-miR-143-3p^25^,^26^ in cancer, these two miRNAs were selected as candidate miRNAs for this study (Fig. 1a).

### hsa-miR-128-3p and hsa-miR-143-3p suppress CYP2C9 3′-UTR luciferase reporter

To test the potential effects of hsa-miR-128-3p and hsa-miR-143-3p on *CYP2C9* expression, a reporter construct retaining the core region of the *CYP2C9* 3′-UTR that harbors the putative binding sites for hsa-miR-128-3p and hsa-miR-143-3p was constructed and then co-transfected into HepG2 and 293T cells together with the hsa-miR-128-3p mimic, hsa-miR-143-3p mimic, or miRNA negative control. Fig. 1b showed that high concentration of exogenous hsa-miR-128-3p (the amount of exogenous hsa-miR128-3p was evaluated by RT-PCR, for which please see Supplementary Fig. S1) efficiently suppressed luciferase activity in HepG2 and 293T cells (34% and 33%, respectively; all *P* < 0.001) as compared with the transfection of miRNA negative control. In contrast, hsa-miR-143-3p exhibited a similar significant suppression effect in 293T cells only (31%, *P* < 0.001), while the effect in HepG2 cells was marginally significant (14%, *P* = 0.062).

To investigate the specificity of hsa-miR-128-3p suppressing *CYP2C9* 3′UTR, CYP2C9-MUT1-CU and CYP2C9-MUT2-CU constructs (with mutated hsa-miR-128-3p targeting sequences in the *CYP2C9* 3′UTR), were created and applied in the reporter gene assays. As shown in Fig. 1c, transfection with the hsa-miR-128-3p mimic did not suppress luciferase activity from CYP2C9-MUT1-CU or CYP2C9-MUT2-CU construct, indicating that hsa-miR-128-3p modulates *CYP2C9* expression in a sequence specific manner by targeting its non-mutated 3′UTR.

### Interaction between CYP2C9 3′-UTR and hsa-miR-128-3p or hsa-miR-143-3p

**Figure 2:**
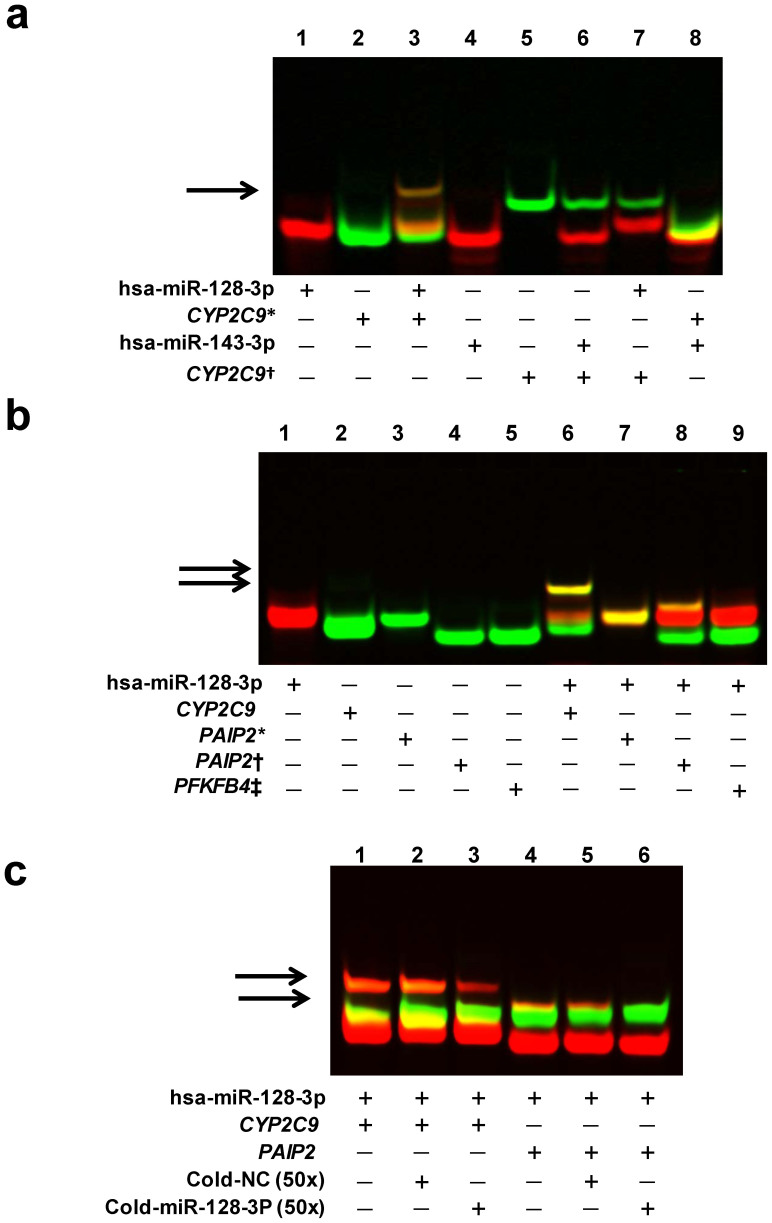
Free energy directly affected the interaction between hsa-miR-128-3p and its counterparts. (a) RNA EMSA with the cy5.5TM-labeled hsa-miR-128-3p or hsa-miR-143-3p oligonucleotides, and 2′ O-Methyl modified and IRDye®800-labeled *CYP2C9* mRNA oligonucleotides. *Lanes 1*, *2*, *4*, and *5* show mobility of the labeled oligonucleotides; *Lanes* *3* and 6 show mobility of the labeled miRNA oligonucleotides with corresponding *CYP2C9* mRNA oligonucleotides; *Lanes* *7* and *8* show mobility of the labeled miRNA oligonucleotides with unmatched *CYP2C9* mRNA oligonucleotides (negative controls). Arrow indicates an oligonucleotide complex (*yellow*) in *Lane 3*. * or † indicates the *CYP2C9* mRNA oligonucleotides retaining the hsa-miR-128-3p or hsa-miR-143-3p recognition sites, repectively. (b) RNA EMSA with the cy5.5TM-labeled hsa-miR-128-3p probe, and 2′ O-Methyl modified and IRDye®800-labeled *CYP2C9*, *PAIP2*, or *PFKFB4* mRNA oligonucleotides. *Lanes 1*, *2*, *3*, *4*, and *5* show mobility of the labeled oligonucleotides; *Lanes* *6*, *7*, *8* and *9* show mobility of the labeled hsa-miR-128-3p oligonucleotide with corresponding *CYP2C9*, *PAIP2*, or *PFKFB4* mRNA probes with different free energy. Arrows indicate the oligonucleotide complex (*yellow*) in *Lane 6* and *8*. *, †, and ‡ indicate two *PAIP2* mRNA oligonucleotides, and one *PFKFB4* mRNA oligonucleotide with the binding free energy of −18.0 kcal/mol, −20.5 kcal/mol, and −15.8 kcal/mol, respectively. (c) RNA EMSA with the cy5.5TM-labeled hsa-miR-128-3p probe, and 2′ O-Methyl modified and IRDye®800-labeled *CYP2C9* or *PAIP2* mRNA oligonucleotides. *Lanes 1* and *4* show mobility of the labeled hsa-miR-128-3p oligonucleotide with corresponding *CYP2C9*, and *PAIP2* mRNA probes with the free energy of −23.9 kcal/mol and −20.5 kcal/mol, respectively. *Lanes 2*, *3*, *5* or *6* show mobility of the labeled hsa-miR-128-3p oligonucleotide with corresponding *CYP2C9* and *PAIP2* mRNA probes, in the presence of unlabeled excess nonspecific competitor (Cold-NC) and excess specific competitor (Cold hsa-miR-128-3p), respectively. NC, nonspecific competitor. Arrows indicate the oligonucleotide complex (yellow) in *Lane* *1*, *2*, *3*, *4* and *5*.

*In silico* analysis predicted that hsa-miR128-3p and hsa-miR143-3p might form complexes with target sequences present in the 3′-UTR of *CYP2C9* to yield unique structures with different calculated free energies of binding: −23.9 kcal/mol for hsa-miR128-3p (Supplementary Fig. S2a) and −16.1 kcal/mol for hsa-143-3p (Supplementary Fig. S2b). To determine whether or not hsa-miR-128-3p or hsa-miR-143-3p is able to bind its cognate *CYP2C9* 3′-UTR mRNA sequence *in vitro*, RNA EMSA was performed. Fig. 2a shows that hsa-miR-128-3p was able to bind the corresponding target sequence of *CYP2C9* 3′-UTR (*lane 3*) and competition assays showed the binding is sequence-specific (*lanes 7*, *8*). There was no evidence of an interaction between *CYP2C9* 3′-UTR and hsa-miR-143-3p (Fig. 2a).

Compared to the related hsa-miR-143-3p complex, the enhanced thermodynamic stability predicted for the hsa-miR-128-3p complex with its *CYP2C9* 3′-UTR target sequence correlated with results from RNA EMSA experiments, confirming the *in vitro* stability of the latter complex only. To explore the applicability of this predictive strategy further, we calculated the free energy of binding between hsa-miR-128-3p and 3 other putative target sequences (2 probes from the *PAIP2* 3′-UTR and 1 probe from the *PFKFB4* 3′-UTR). The predicted free energies of binding were −20.5 kcal/mol, −18.0 kcal/mol, and −15.8 kcal/mol for miR-128-PAIP2-target2, miR-128-PAIP2-target1 and miR-128-PFKFB4-target, respectively. EMSA assays were then conducted with these miRNAs and their targets. The hsa-miR-128-3p mimic was able to bind the probe with the free energy of −20.5 kcal/mol (Fig. 2b, *lane 8*), but not the ones with free energy of −18.0 kcal/mol, or −15.8 kcal/mol (Fig. 2b, *lane 7* or *9*), showing a correlation between the predicted free energy of binding directly and the observed interaction between miRNAs and their counterparts, which we propose should also correlate with the efficiencies of gene regulation by these miRNAs.

In addition, we found that the miRNA-mRNA complex, formed by hsa-miR-128-3p with *CYP2C9* 3′UTR, or with *PAIP2* 3′UTR, could be eliminated by adding 50× unlabeled hsa-miR-128-3p probe(Fig. 2c, *lane 3* or *6*), but not by adding a nonspecific probe (Cold-NC) (Fig. 2c, *lane 2* or *5*), suggesting the binding between hsa-miR-128-3p and its cognate 3′UTR of *CYP2C9* or *PAIP2* is in a sequence-specific manner.

### Suppression of endogenous CYP2C9 expression/translation by exogenous hsa-miR-128-3p

**Figure 3:**
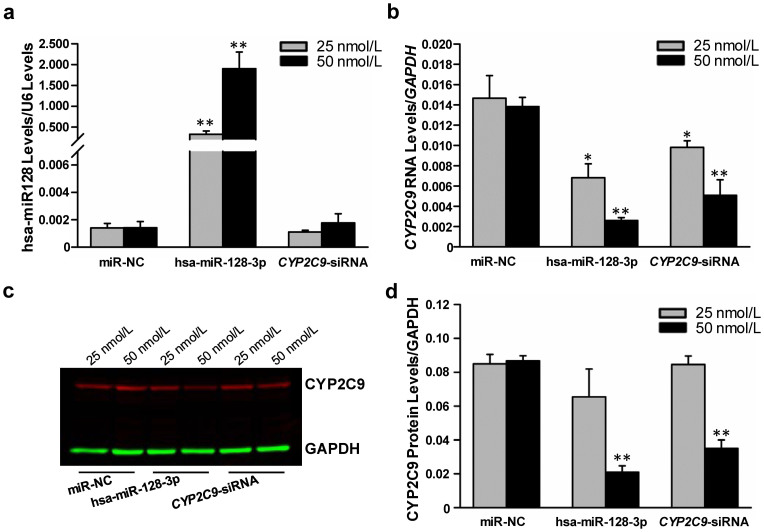
The hsa-miR-128-3p suppressed endogenous *CYP2C9* expression in HepaRG cells. Differentiated HepaRG cells were transiently transfected with 25 nmol/L or 50 nmol/L hsa-miR-128-3p mimic, CYP2C9-specific siRNA, or miRNA negative control, respectively, and harvested 48 hours after transfection. Each assay was done on at least 3 independent transfection experiments. NC, miRNA negative control; * *P* < 0.05; ** *P* < 0.001. (a) Unregulated expression of hsa-miR-128-3p. Data were shown as relative hsa-miR-128-3p levels versus U6. (b) Down-regulated expression of *CYP2C9* mRNA by hsa-miR-128-3p or *CYP2C9*-specific siRNA. Data are shown as relative *CYP2C9* mRNA levels versus *GAPDH* reference. (c) and (d) Down-regulated CYP2C9 protein levels by hsa-miR-128-3p or CYP2C9-specific siRNA.

It was reported that HepaRG cells express CYP2C9 at a level similar to that found in primary hepatocytes, while the HepG2 cells barely express CYP2C9 owing to the lack of nuclear factors and other transcription factors for the expression of DMEs^27^. We did observed that the inherent *CYP2C9* mRNA level in HepaRG cells was ~10-fold and ~20-fold greater than those in 293T cells and HepG2 cells, respectively, while the hsa-miR-128-3p was expressed in a similar level among these cell lines (Supplementary Fig. 3a). To investigate the effects of hsa-miR-128-3p on endogenous *CYP2C9* transcripts and protein levels, hsa-miR-128-3p mimics, miRNA negative control or a *CYP2C9*-specific siRNA that served as the positive control, were transiently transfected into HepaRG cells. Fig. 3a shows that the level of hsa-miR-128-3p was dramatically increased by more than 200-fold after transfection with hsa-miR-128-3p mimics at concentrations of 25 nmol/L or 50 nmol/L. Consequently, *CYP2C9* mRNA expression was significantly decreased compared to that in cells transfected with the miRNA negative control (53.7% at 25 nmol/L and 81.1% at 50 nmol/L; all *P* < 0.05) (Fig. 3b). In addition, the CYP2C9 protein level decreased significantly (76.7%) when the cells were transfected with 50 nmol/L hsa-miR-128-3p mimics in comparison with the transfection of the miRNA negative control (Fig. 3c and 3d). Notably, transfection of hsa-miR-128-3p was more efficient at suppressing CYP2C9 protein expression than transfection of the *CYP2C9*-specific siRNA positive control (Fig. 3b, 3c and 3d).

*PTEN* was proved to be negatively associated with hsa-miR-128-3p level in pituitary cells, probably due to hsa-miR-128-3p targeting *BMI1*, one transcriptional suppressor of *PTEN*^28^. As a positive control, we observed that the *PTEN* mRNA level was decreased significantly after hsa-miR-128-3p transfection, compared with that in cells transfected with the miRNA negative control (40.1% at 25 nmol/L and 46.6% at 50 nmol/L; all *P* < 0.05) (Supplementary Fig. S4a), suggesting that our transfection experimental system is validated.

### Modulation of CYP2C9 expression/translation through chemically-induced alteration of hsa-miR-128-3p levels

**Figure 4:**
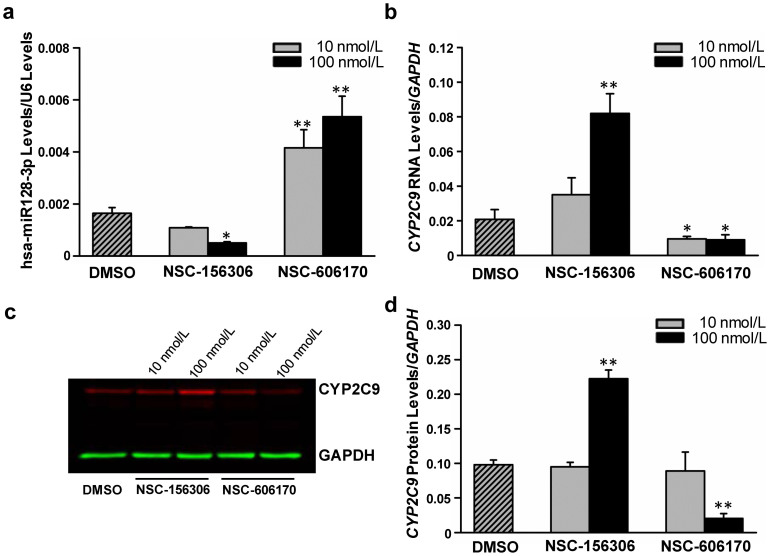
Chemical compounds NSC-156306 and NSC-606170 affected endogenous *CYP2C9* expression by altering the expression of hsa-miR-128-3p in HepaRG cells. Differentiated HepaRG cells were treated with 0, 10 nmol/L, or 100 nmol/L NSC-156306 or NSC-606170, and harvested 48 hours after treatments. Each assay was performed from at least 3 independent experiments. * *P* < 0.05; ** *P* < 0.001. (a) Dysregulated expression of hsa-miR-128-3p. Data are shown as relative hsa-miR-128-3p levels versus U6. (b) Dysregulated expression of *CYP2C9* mRNA by NSC-156306 or NSC-606170 treatment. Data are shown as relative *CYP2C9* mRNA levels versus *GAPDH* reference. (c) and (d) Dysregulated CYP2C9 protein levels by NSC-156306 or NSC-606170 treatment.

According to the CellMiner™ database (version 1.5, http://discover.nci.nih.gov/cellminer), chemical compounds NSC-156306 and NSC-606170 appear to inhibit or induce, respectively, the expression of hsa-miR-128 in human liver tissue. Therefore, we treated HepaRG cells with these compounds to determine their impact on *CYP2C9* transcription and translation. Fig. 4 shows that treatment of the HepaRG cells with 100 nmol/L NSC-156306 resulted in a significant decrease in the expression of hsa-miR-128-3p and that its level was markedly increased after treating cells with NSC-606170 (Fig. 4a). The treatment-induced hsa-miR-128-3p expression changes were accompanied by inverse alterations in the expression of *CYP2C9* gene expression (Fig. 4b) and protein production (Fig. 4c and 4d). Specifically, the levels *CYP2C9* mRNA and protein in the NSC-156306-treated HepaRG cells were 3-fold and ~2-fold greater (Fig. 4b, 4c and 4d). In contrast, the levels of *CYP2C9* mRNA and protein were dramatically decreased, by 81.1% and 79.6%, in cells treated with NSC-606170 (Fig. 4b, 4c and 4d). In addition, we also observed that the *PTEN* mRNA level was decreased significantly after NSC-606170 treatment in HepaRG cells (67.6% at 10 nmol/L and 75.3% at 100 nmol/L; all *P* < 0.05) (Supplementary Fig. S4b), suggesting that the modulation of hsa-miR-128-3p by NSC-606170 was obtained.

### Inverse correlation between hsa-miR-128-3p and *CYP2C9* expression in HCC

**Figure 5:**
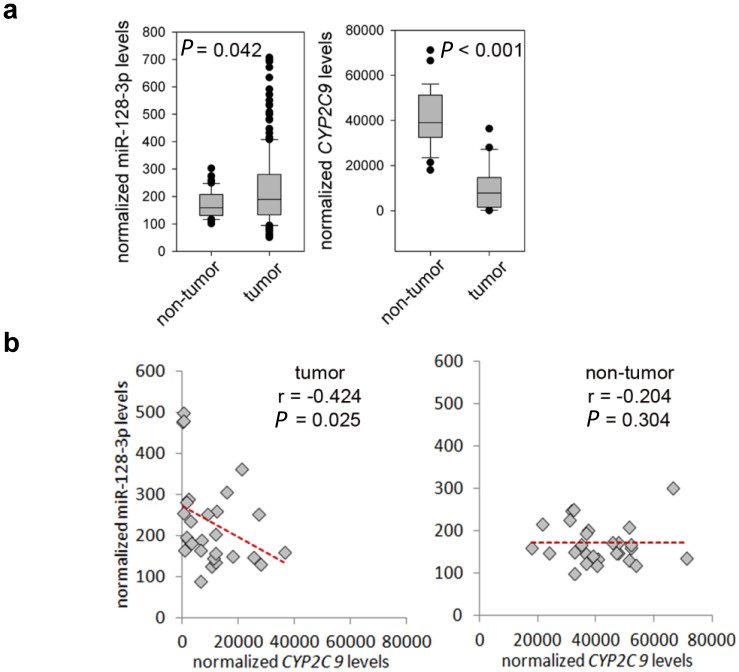
Relationship between *CYP2C9* mRNA expression and hsa-miR-128-3p level in HCC and paired non-tumor tissues. (a) Expression data from HCC tumor and non-tumor samples stored in The Cancer Genome Atlas (TCGA) was downloaded and compared using the Rank Sum Test. There were 28 and 27 tumor and non-tumor samples respectively that had expression data for hsa-miR-128-3p and *CYP2C9*. Data has been normalized to reads per million. (b) hsa-miR-128-3p levels correlate with *CYP2C9* levels in tumor tissues (r = −0.424, *P* = 0.025, 28 matched tumor samples), but there is no significant correlation in the non-tumor tissues (r = −0.204, *P* = 0.304, 27 matched non-tumor samples).

The expression levels of hsa-miR-128-3p and CYP2C9 mRNA in human HCC tumor tissue samples and adjacent normal liver samples were extracted from The Cancer Genome Atlas (TCGA) database. Levels of hsa-miR-128-3p measured in tumor tissues were significantly higher than those measured in matched non-tumor tissues; whereas, levels of *CYP2C9* were significantly reduced in tumor tissues compared to surrounding non-tumor tissues (Fig. 5a). The relationship between hsa-miR-128-3p and *CYP2C9* expression was evaluated by the Spearman Rank Order Correlation analysis in patient matched tumor and non-tumor tissues. In tumor tissues, there is a negative correlation between hsa-miR-128-3p and *CYP2C9* (r = −0.424, *P* = 0.025, Fig. 5b), but in non-tumor tissues there is no significant correlation (r = −0.204, *P* = 0.304, Fig. 5b). Interestingly, no statistically significant correlation between *PTEN* mRNA (as a known miR-128 target) levels and hsa-miR-128-3p levels was observed in those tissues (Supplementary Fig. S5), probably due to the complexity and high heterogeneity of gene expression in tumor tissue. When hsa-miR-143-3p was examined, no statistically significant results were obtained (Data not shown).

## Discussion

Inter-individual variability in the expression of DMEs in humans is an important phenotypic trait that may contribute significantly to disparities in disease susceptibility and drug efficacy^29^,^30^,^31^. The mechanism underlying the phenotype is due, to a certain extent, to genetic polymorphisms and epigenetic variation among human populations. Genetic polymorphism(s) could alter gene transcription or change an enzyme's catalytic activity, while miRNAs regulate gene expression by targeting the mRNAs, repressing protein translation or accelerating mRNA degradation. In this study, we demonstrated that hsa-miR-128-3p plays an important role in the suppression of CYP2C9 expression and translation in human liver cells by a series of *in silico* analyses and *in vitro* and *in vivo* experiments. This study helped to elucidate the functional mechanism by which miRNA regulates CYP2C9 expression and translation. In addition, a modified RNA EMSA method provided direct evidence for the interaction between miRNAs and their cognate mRNA sequences that was dependent on the predicted free energy of binding.

CYP2C9 is one of the most abundant and important xenobiotic metabolizing enzymes, with substrates including commonly prescribed drugs such as warfarin, NSAIDs (non-steroidal anti-inflammatory drugs), tolbutamide, phenytoin, and torasemide^13^. CYP2C9 is also reported to participate in the bioactivation of carcinogens. For example, metabolism of benzo[a]pyrene by CYP2C9 results in the formation of 9-hydroxybenzo[a]pyrene-4,5-oxide and benzo[a]pyrene-7,8-diol-9,10-epoxide, which are reactive species involved in DNA adduct formation^32^,^33^,^34^. In addition, a high activity *CYP2C9* genotype (CYP2C9*1) is associated with increased risk of colorectal cancer^35^ while a low activity *CYP2C9* genotype (CYP2C9*2) is associated with increased risk of colorectal adenoma^36^ and lung cancer^37^. Furthermore, decreased *CYP2C9* expression was reported in HCC tissue by several studies^14^,^15^,^16^,^17^, suggesting the role of CYP2C9 in detoxification may be involved in the etiology of HCC; however, the mechanisms controlling *CYP2C9* expression are not fully understood.

In this study, we demonstrated that hsa-miR128-3p plays a pivotal role in the regulation of *CYP2C9* expression in human liver using series of *in silico*, *in vitro*, and *in vivo* analyses. It is well known that the interaction between miRNAs and their cognate mRNA targets is complicated, and there are more than ten algorithms for predicting miRNA targets^38^. Therefore, for a given miRNA or transcript, many “putative” or “potential” interactions are predicted by *in silico* approaches. To reduce false positive predictions and validate true interactions, we first screened the miRNAs that could potentially target the 3′-UTR of *CYP2C9* mRNA using the microRNA.org database, and then evaluated the resultant candidate miRNAs in the PITA and TargetScan databases. The candidate miRNAs that were predicted by all three database algorithms to target *CYP2C9* mRNA were then experimentally examined by both *in vivo* and *in vitro* approaches for functional interaction with *CYP2C9* mRNA. The selection of hsa-miR-128-3p and hsa-miR-143-3p as candidate miRNAs for further biochemical characterization was based on their reported biological significance in tumorigenesis and metastasis^22^,^23^,^24^,^25^,^26^. For example, hsa-miR-128 is aberrantly expressed in many types of tumors, including acute lymphoblastic leukemia, glioblastoma, and breast cancer. By targeting *EGFR*, *Bim-1*, *ABCC5* and other genes, hsa-miR-128 is involved in tumor differentiation, proliferation, invasion, apoptosis and resistance to drugs^39^. The miRNA hsa-miR-143 was reported as a tumor suppressor in cervical cancer^40^ and prostate cancer^41^, by suppressing *KRAS*, *ERK5*, and other genes.

We first transfected the hsa-miR-128-3p or hsa-miR-143-3p mimics into liver HepG2 and kidney 293T cells, together with a reporter gene (luciferase) plasmid containing the core region of *CYP2C9* 3′-UTR, and found that hsa-miR-128-3p suppressed luciferase activity in both liver cells and kidney cells, while hsa-miR-143-3p exhibited a relatively smaller suppression effect only in kidney cells. RNA EMSA assays revealed that hsa-miR-128-3p bound *CYP2C9* mRNA, while an interaction between hsa-miR-143-3p and the *CYP2C9* 3′-UTR was not detected. Further, we used RNA EMSA to test the binding efficiencies between hsa-miR-128-3p and three other target sequences with different free energies of binding predicted by the RNAhybrid software. It was observed that only the probes with free energy of less than −20 kcal/mol could bind hsa-miR-128-3p under our experimental conditions. Therefore, we postulate that the binding efficiency between miRNAs and their cognate mRNA targets is mainly dependent on the free energy state of the binding. Althogh further studies on the precise mechanisms of miRNA targeting mRNA sequences are under way, our current results provide evidence that the free energy of binding is important for accurate predictions of miRNA targeting sites.

Because of the low expression of DMEs and transporters by HepaG2 and other hepatocellular carcinoma cell lines^30^,^42^, we used HepaRG cells, which express DMEs and transporters at levels similar to primary hepatocytes, to investigate the suppression effects of hsa-miR-128-3p on endogenous CYP2C9 expression and translation. Our results showed that enforced up-regulattion of hsa-miR-128-3p reduced CYP2C9 production at both the protein and mRNA levels, indicating that hsa-miR-128-3p is at least involved in *CYP2C9* mRNA degradation. Two chemicals, with strong negative or positive effect on hsa-miR-128 expression, were used to produce CYP2C9 alterations through the modulation of hsa-miR-128 expression. The results confirmed that the dramatic changes in hsa-miR-128-3p expression caused by these two compounds (Fig. 4a) indeed altered the CYP2C9 expression/production inversely (Fig. 4b, 4c and 4d), which is consistent with the transfection assays. Altogether, our results indicated that hsa-miR-128-3p is able to suppress CYP2C9 expression/production in human hepatic cells by specifically targeting the 3′-UTR of CYP2C9 mRNA molecules.

Finally, the correlation between the expression of *CYP2C9* and hsa-miR-128-3p in HCC tissues was evaluated using the GSE22058 and TCGA datasets. We observed that *CYP2C9* mRNA was significantly down-regulated in HCCs, consistent with findings reported by others^14^,^15^,^16^,^17^. Most importantly, this study revealed an up-regulation of hsa-miR-128-3p expression and a significant inverse correlation between *CYP2C9* expression and hsa-miR-128-3p expression in HCC tissues, indicating that hsa-miR-128-3p combined with *CYP2C9* are potential biomarkers for HCC diagnosis.

In summary, our study identified and experimentally confirmed that hsa-miR-128-3p is a suppressor for *CYP2C9* expression in HCC, and revealed a direct interaction between a miRNA and its target mRNA sequence *in vitro*, which demonstrated a molecular mechanism of miRNA mediated *CYP2C9* suppression.

## Methods

### Cell lines

HepG2 and 293T cells were obtained from the American Type Culture Collection (ATCC, Manassas, VA) and HepaRG cells were obtained from Biopredic International (Overland Park, HS). The cells were maintained according to ATCC and Biopredic International's recommendations.

### *In silico* analyses

The public databases microRNA.org (http://www.microrna.org/), PITA (http://genie.weizmann.ac.il/pubs/mir07/mir07_prediction.html) and TargetScan (Release 6.2, http://www.targetscan.org) were screened to identify potential miRNA response elements (MREs) located in the 3′-UTR of the *CYP2C9* gene. The RNAhybrid program^43^ was used to calculate the free energies of binding for predicted miRNA:RNA duplexes formed between hsa-miR-128-3p and *CYP2C9*, *PAIP2* or *PFKFB4* mRNA sequences. The CellMiner™ database (version 1.5, http://discover.nci.nih.gov/cellminer), which integrates the molecular and pharmacological data sets for the NCI-60 cell lines, was used to select the chemical compounds that have demonstrated positive or negative correlations with hsa-miR-128-3p expression.

### Luciferase reporter gene assay

The pGL3-Control vector (Promega, Madison, WI) was modified by adding the Universal USER Cassette (New England Biolabs, Ipswich, MA), similar to the modification of the pGL3-Promoter^44^. Briefly, the Xba I site was digested and blunted by using the Quick Blunting Kit (New England Biolabs). The USER Cassette sequence was then inserted, resulting in the pGL3-CU vector. Cloning primers CYP2C9-F and CYP2C9-R (All primer or oligo sequences used in this study were listed in Supplementary Table S1), with extension oligonucleotides 5′-GGA GAC AU-3′ or 5′-GGG AAA GU-3′ in their 5′ end, were designed to amplify the core region of *CYP2C9* 3′-UTR that harbors the putative binding sites for hsa-miR-128-3p and hsa-miR-143-3p. PCR products were digested with USER enzyme (New England Biolabs) and cloned into the linearized nicked pGL3-CU vector that was prepared following the Universal USER Cassette protocol. The DNA sequence of the resultant plasmid, designated CYP2C9-CU, was determined to confirm its identity. Besides, CYP2C9-MUT1-CU or CYP2C9-MUT2-CU construct, which with mutated hsa-miR-128-3p target sequences in the *CYP2C9* 3′UTR, was created by site-directed mutagenesis using CYP2C9-MUT1-F and CYP2C9-MUT1-R primers, or CYP2C9-MUT2-F and CYP2C9-MUT2-R primers, respectively.

HepG2, a human hepatoma cell line, and 293T, a human embryonic kidney line, were used for luciferase assays. HepG2 or 293T cells were cultured in Rosewell Park Memorial Institute 1640 or Dulbecco's Modified Eagle medium with 10% fetal bovine serum. Cells were seeded in 96-multiwell plates. When the cells reached 70%–80% confluence, they were transfected with the CYP2C9-CU plasmid (100 ng/well) that contains the 3′-UTR of CYP2C9 together with 50 nmol/L (final concentration) hsa-miR-128-3p mimic, hsa-miR-143-3p mimic, or miRNA negative control (Thermo Scientific, Tewksbury, MA) using the Lipofectamine reagent 2000 (Life Technologies, Carlsbad, CA). The pRL-SV40 (1 ng/well; Promega) plasmid, which expresses *Renilla reniformis* luciferase, was co-transfected to standardize transfection efficiency. The CYP2C9-CU plasmid co-transfected with pRL-SV40 plasmid and miRNA negative control served as a reference. Three independent transfection experiments were carried out, and each transfection was performed in triplicate.

### RNA electrophoretic mobility shift assay (EMSA)

The miRNA oligonucleotides hsa-miR-128-3p and hsa-miR-143-3p were labeled with cy5.5™ dye on their 5′ ends. The 2′ *O*-methyl-modified mRNA oligonucleotides miR-128-CYP2C9-target, miR-128-PAIP2-target1, miR-128-PAIP2-target2, miR-128-PFKFB4-target, and miR-143-CYP2C9-target were labeled with IRDye®800 (LI-COR Biosciences, Lincoln, NE) dye on their 5′ ends. All primers and oligonucleotides used in this study were synthesized by Integrated DNA Technologies (Coraville, IA).

RNA EMSAs were performed according to the LightShift Chemiluminescent RNA EMSA Kit (Thermo Scientific) protocol. Briefly, in each 20 μL binding reaction containing 200 nmol synthetic miRNA or/and cognate mRNA binding oligonucleotides were mixed, heated for 5 minutes at 80°C, placed on ice to relax RNA secondary structures, and then incubated at 25°C for 20 min. The reaction mixtures were separated on a 12% PAGE by electrophoresis at 4°C, and the resultant mobility shifts were detected with an Odyssey CLx Infrared Imaging System (LI-COR Biosciences, Lincoln, NE).

### Transfection of HepaRG cells with hsa-miR-128-3p and treatments with chemical compounds

HepaRG cells (Biopredic International, Overland Park, KS) were first incubated in Williams' E medium supplemented with growth supplement (Biopredic International) for 2 weeks and then cells were differentiated by adding the differentiation supplement (Biopredic International) for 10 additional days. Differentiated cells were then seeded into 6-well plates at a density of 70,000 cells/cm^2^ with 3 ml medium and incubated for another 2 days for further experiments.

The miRNA transfection was performed as previously reported^45^. Briefly, 25 nmol/L or 50 nmol/L (final concentration) hsa-miR-128-3p mimic, miRNA negative control, or CYP2C9-specific siRNA (positive control), was transfected into the differentiated HepaRG cells using Lipofectamine transfection reagent (Life Technology), and cells were harvested 48 hours after transfection. Chemical compounds with the NSC numbers NSC-156306 (*o*-amsa monomethanesulfonate) and NSC-606170 (zalcitabine) were obtained from the Developmental Therapeutics Program (DTP) of the National Cancer Institute (NCI) and diluted to 20 μmol/L using dimethyl sulfoxide (DMSO). Differentiated HepaRG cells were treated with 0, 10, or 100 nmol/L (final concentration) of NSC-156306 or NSC-606170 and cells were harvested 48 hours after treatment. Each assay was conducted using at least three independent transfection experiments or chemical treatment.

### RNA isolation and quantitative reverse-transcription PCR (qRT-PCR)

Total RNA was extracted from HepaRG cells after transfection or chemical treatment using the miRNeasy Mini Kit (Qiagen, Valencia, CA) and cDNA was synthesized using QuantiTect Reverse Transcription Kit (Qiagen) or NCode™ miRNA First-Strand cDNA Synthesis Kit (Life Technologies). *CYP2C9*, *PTEN* and *GAPDH* RNA levels were measured by qRT-PCR on an ABI Prism7900 Sequence Detection System (Applied Biosystems) according to the QuantiFast SYBR® Green RT-PCR Kit (Qiagen) protocol using the CYP2C9-RT-F and CYP2C9-RT-R primers, PTEN-RT-F and PTEN-RT-R primers, or GAPDH-RT-F and GAPDH-RT-R primers, respectively. The miR-128-RT-F and U6-F primers, together with the Universal Reverse Primer supplied with the NCode™ miRNA First-Strand cDNA Synthesis Kit, were used to detect the hsa-miR-128-3p and *U6* levels. The RNA expression levels of *CYP2C9* or hsa-miR-128-3p were calculated relative to expression of *GAPDH* or *U6*, respectively.

### Western blot analysis

Proteins were isolated from HepaRG cells that were harvested after transfection or chemical treatment. Quantitative Western blotting was performed following the Odyssey™ Western Blotting Kit (LI-COR Biosciences) protocol. Antibodies against CYP2C9 or GAPDH (Abcam, Cambridge, MA) were used to detect CYP2C9 or GAPDH protein levels and an Odyssey CLx Infrared Imaging System was used to perform quantitative analyses, with infrared labeled secondary antibodies.

### Retrieval of data from online databases

RNA expression levels of *CYP2C9* and hsa-miR-128-3p were obtained from The Cancer Genome Atlas database (TCGA, http://cancergenome.nih.gov/).

### Statistical analyses

The rank sum test was used to evaluate the difference in the expression of *CYP2C9* or hsa-miR-128-3p in HCCs, with *P* < 0.05 as the significant criterion. Spearman Rank Order Correlation analysis was used to test the correlation between *CYP2C9* levels and hsa-miR-128-3p expression. Student's *t*-test was also used to compare results from luciferase reporter gene assays and to compare *CYP2C9* or hsa-miR-128-3p protein or RNA levels between subgroups.

## Author Contributions

B.N. proposed and organized the study. D.Y., I.P. and B.N. designed the study. D.Y., B.G. and A.M. performed experiments. D.Y. and B.N. wrote the manuscript. Y.G., S.K., D.L. and J.F. provided advice and revised the manuscript. All authors reviewed the manuscript.

## Supplementary Material

Supplementary Information Supplementary Information
