# Supplementary material for: Suppression of CYP2C9 by MicroRNA hsa-miR-128-3p in Human Liver Cells and Association with Hepatocellular Carcinoma
Source: Sci Rep. 2015 Feb 23;5:8534. doi: 10.1038/srep08534 (PMC4336941; doi:10.1038/srep08534)
Supplement: Supplementary Information [file srep08534-s1.pdf]

Suppression of CYP2C9 by MicroRNA hsa-miR-128-3p in Human Liver Cells and Association with Hepatocellular Carcinoma

Dianke Yu[1, 2], Bridgett Green[1], April Marrone[1], Yongli Guo[3], Susan Kadlubar[4], Dongxin Lin[2], James Fuscoe[1], Igor Pogribny[1] and Baitang Ning[1]*

[1]National Center for Toxicological Research, US Food and Drug Administration, Jefferson, AR 72079, USA

[2]State Key Laboratory of Molecular Oncology and Department of Etiology & Carcinogenesis, Cancer Institute and Hospital, Chinese Academy of Medical Sciences and Peking Union Medical College, Beijing, China 100021

[3]Beijing Children's Hospital, Capital Medical University, Beijing, China 100045

[4]University of Arkansas for Medical Sciences, AR 72205, USA

*Correspondence and requests for materials should be addressed to
Baitang Ning, Ph.D.

National Center for Toxicological Research

3900 NCTR Road, HFT100

Jefferson, AR 72079

Phone: 870-543-7129

Fax: 870-543-7773

Email: baitang.ning@fda.hhs.gov

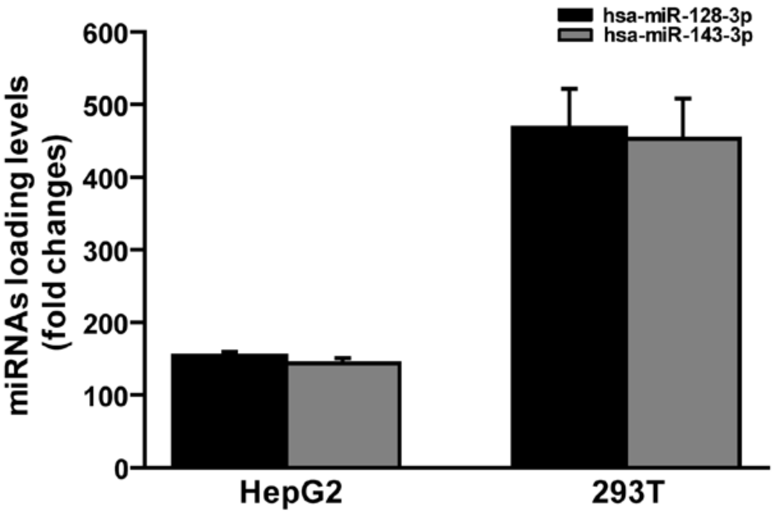

Supplementary Figure S1. The loading levels of hsa-miR-128-3p or hsa-miR-143-3p after miRNA mimics transfection in reporter gene assays.

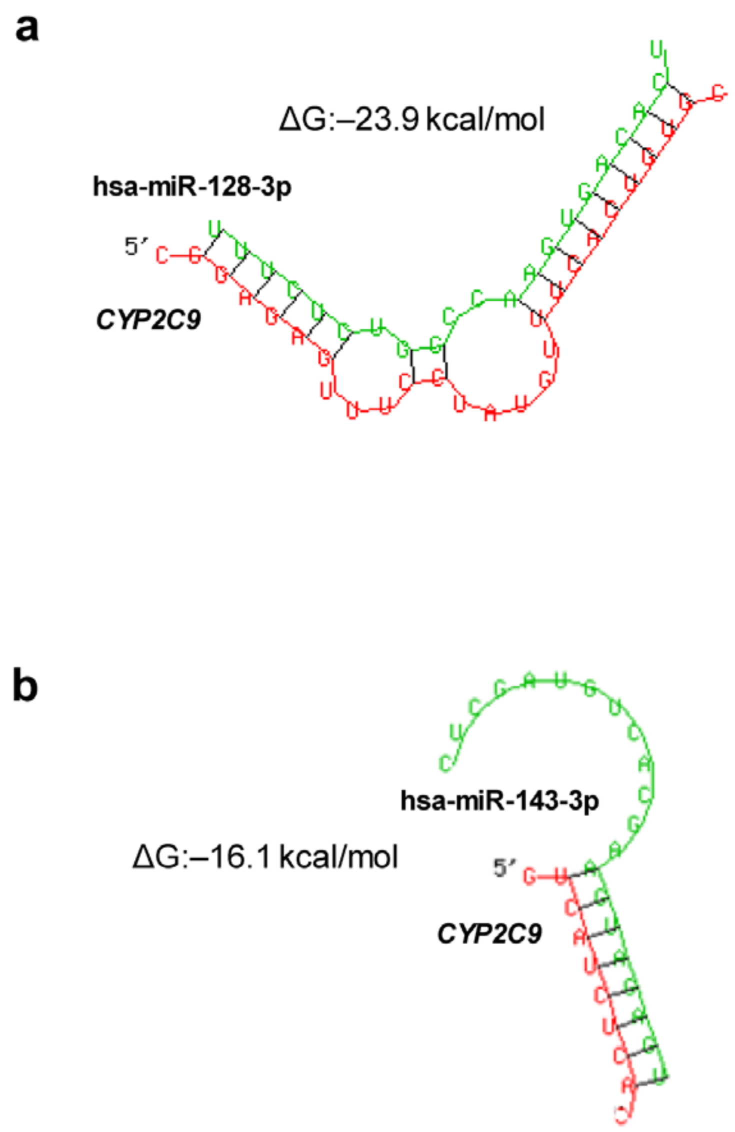

Supplementary Figure S2. Free energy analyses of the interaction between hsa-miR-128-3p or hsa-miR-143-3p with *CYP2C9* 3'-UTR. (a) Free energy analysis of the pairing of hsa-miR-128-3p to the recognition site of *CYP2C9* 3'-UTR. ΔG, free energy. (b) Free energy analysis of the pairing of hsa-miR-143-3p to the recognition site of *CYP2C9* 3'-UTR.

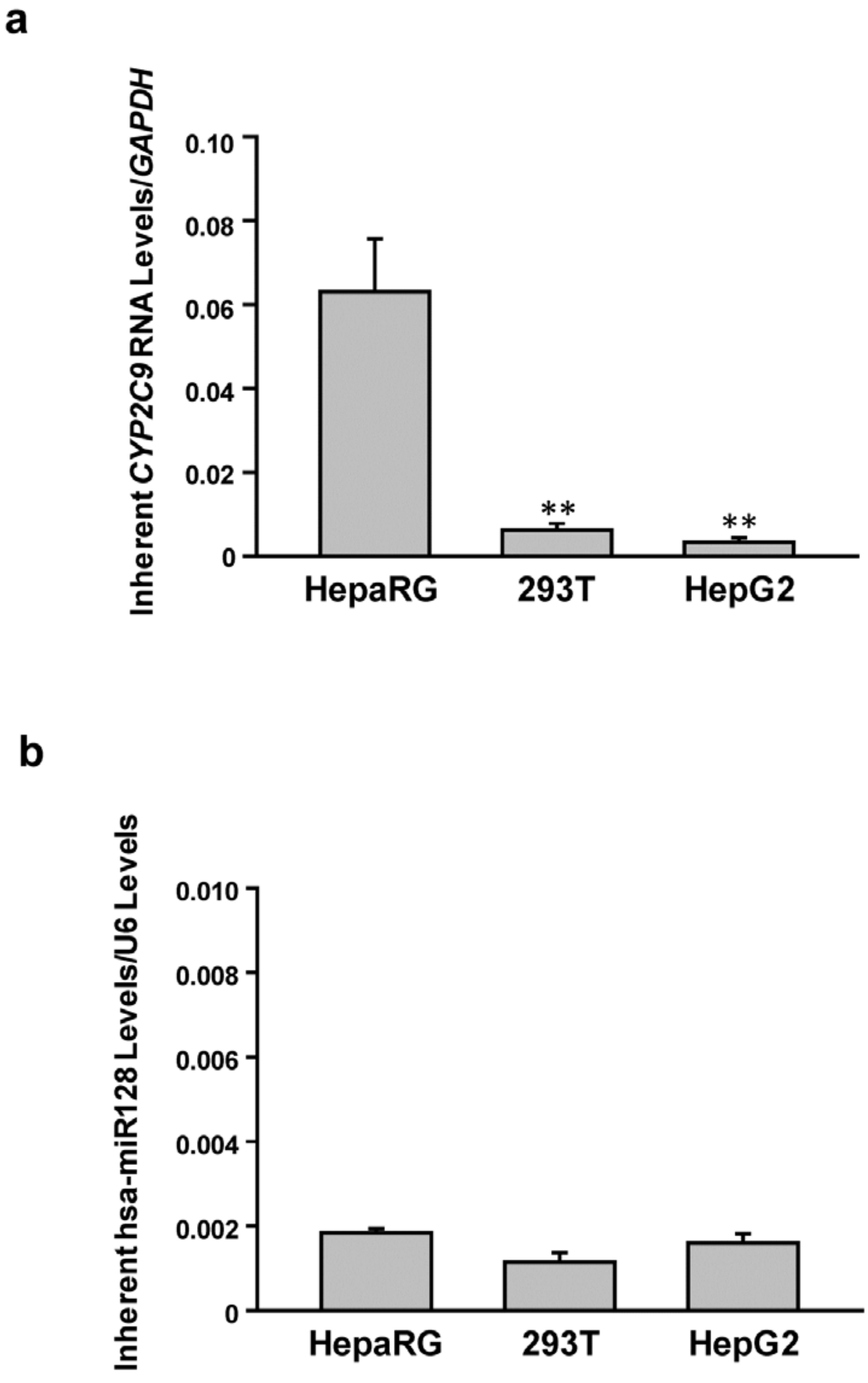

Supplementary Figure S3. Inherent (a) *CYP2C9* and (b) hsa-miR-128-3p RNA Levels in HepaRG, 293T and HepG2 cells. ** P<0.001.

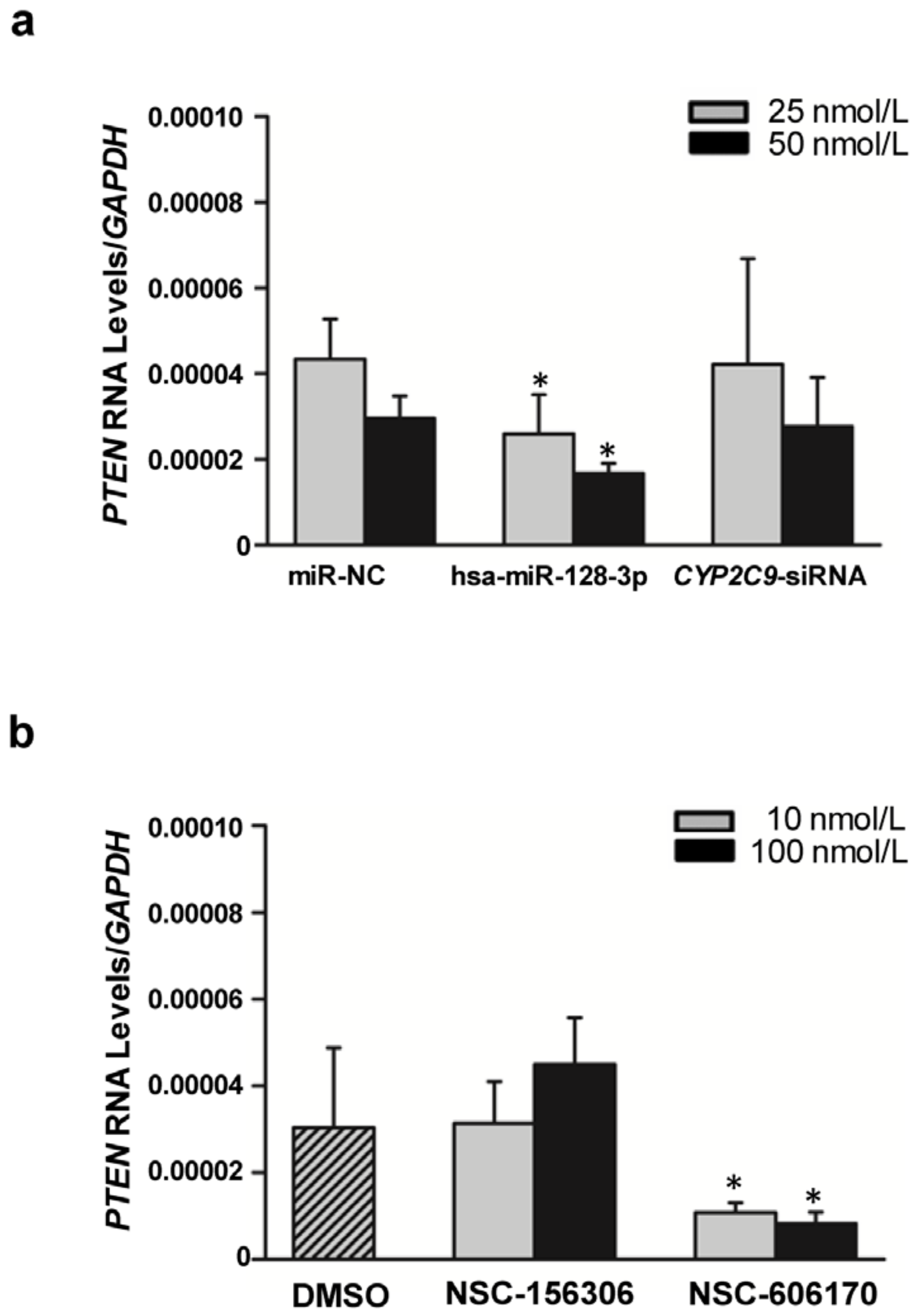

Supplementary Figure S4. The (a)hsa-miR-128-3p and (b) chemical compound NSC-606170 suppressed endogenous *PTEN* expression in HepaRG cells. Each assay was performed from at least 3 independent experiments. * $P<0.05$.

a

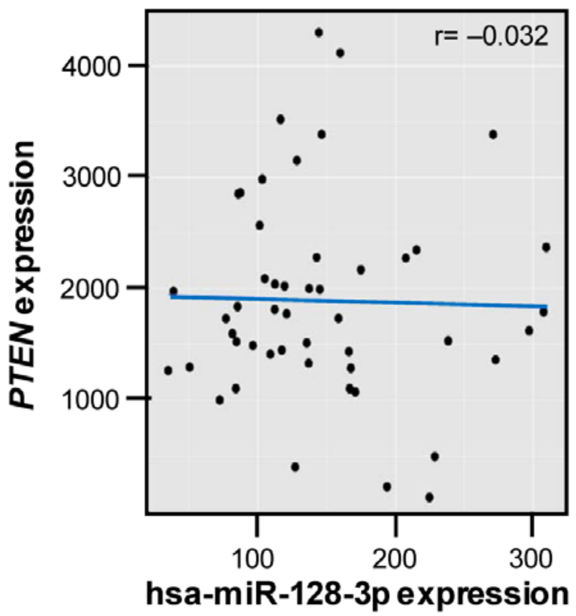

b

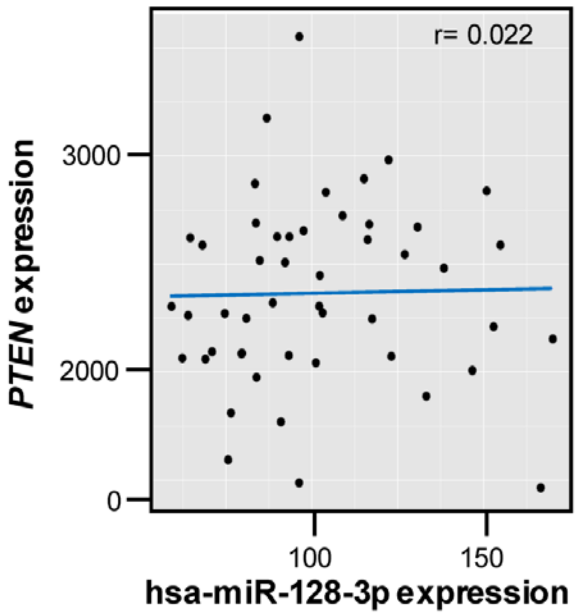

Supplementary Figure S5. Relationship between *PTEN* mRNA expression and hsa-miR-128-3p level in (a) HCC and (b) paired non-tumor tissues.

Supplementary Table S1. Primers and Oligos used in the Study

Primers/Oligos	Sequence (5’ to 3’)	Used for
CYP2C9-F	GGGAAAGUAGAAGAGCAGATGGCCTG	Reporter vector construction
CYP2C9-R	GGAGACAUGACTCAGCAAATAATAATGCTTT	Reporter vector construction
CYP2C9-MUT1-F	AGTTTCCTATGTTTCACTATGCAAATATATC	Reporter vector construction
CYP2C9-MUT1-R	GATATATTTGCATAGTGAAACATAGGAAACT	Reporter vector construction
CYP2C9-MUT2-F	AGTTTCCTATGTTTCAATGTGCAAATATATC	Reporter vector construction
CYP2C9-MUT2-R	GATATATTTGCACATTGAAACATAGGAAACT	Reporter vector construction
hsa-miR-128-3p	/cy5.5™/UCACAGUGAACCGGUCUCUUU	RNA EMSA
hsa-miR-143-3p	/cy5.5™/UGA GAU GAA GCA CUG UAG CUC	RNA EMSA
miR-128-CYP2C9-target	/IRDye800/AGAGUUUCCUAUGUUUCACUGUGCAAAUAUAUC	RNA EMSA
miR-128-PAIP2-target1	/IRDye800/UAAAAGCUCUCUUGUCACUGUGU	RNA EMSA
miR-128-PAIP2-target2	/IRDye800/UGCCAUACGUGUUCAGUGUGA	RNA EMSA
miR-128-PFKFB4-target	/IRDye800/UCAGCCACAUGCAACACUGUGU	RNA EMSA
miR-143-CYP2C9-target	/IRDye800/CCUUUUCUCACCUGUCAUCUCACAUUUUCCCUU	RNA EMSA
Cold-miR-128-3p	UCACAGUGAACCGGUCUCUUU	RNA EMSA
Cold-NC	UCACAACCUCCUAGAAAGAGUAGA	RNA EMSA
CYP2C9-RT-F	GACATGAACAACCCTCAGGACTTT	qRT-PCR
CYP2C9-RT-R	TGCTTGTCGTCTCTGTCCCA	qRT-PCR
PTEN-RT-F	CCGAAAGGTTTTGCTACCATTCT	qRT-PCR
PTEN-RT-R	AAAATTATTTCCTTTCTGAGCATTCC	qRT-PCR
GAPDH-RT-F	GAAATCCCATCACCATCTTCCAGG	qRT-PCR
GAPDH-RT-R	GAGCCCCAGCCTTCTCCATG	qRT-PCR
miR-128-RT-F	TCACAGTGAACCGGTCTCTTT	qRT-PCR
U6-F	CTCGCTTCGGCAGCACA	qRT-PCR
